# The big picture: Parasites, People and the Path to Ending Schistosomiasis

**DOI:** 10.1038/s44319-026-00845-x

**Published:** 2026-06-26

**Authors:** Francisca Mutapi, Mark E J Woolhouse

**Affiliations:** 1 Institute of Immunology and Infection Research, Ashworth Laboratories, King’s Buildings, University of Edinburgh, EH93FL Edinburgh, UK; 2 Tackling Infections to Benefit Africa (TIBA) Partnership, Ashworth Laboratories, King’s Buildings, University of Edinburgh, Edinburgh, UK; 3 Usher Institute, Ashworth Laboratories, King’s Buildings, University of Edinburgh, EH93FL Edinburgh, UK

**Keywords:** Immunology, Microbiology, Virology & Host Pathogen Interaction, Pharmacology & Drug Discovery

## Abstract

Affecting 110 million African children, schistosomiasis control demands an urgent shift from drug treatments to permanent transmission interruption. This requires pairing paediatric drug formulations and new interventions including any new vaccines with locally-relevant multisectoral policy, WASH infrastructure and community-led behaviour change as well as sustained domestic financing.

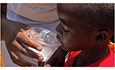

Schistosomiasis is the second most important parasitic disease in Africa, second only to malaria, and the continent carries most of the global burden of this neglected tropical disease (WHO, 2020). Today, an estimated 110 million African children up to 16 years old are affected. If these children stood hand in hand, they would form a human chain long enough to circle the Earth more than three times (NASA, 2023). This striking image underscores the sheer scale of this chronic disease that affects a vast number of children during the most vulnerable years of their lives. It also highlights the urgency of moving beyond treatment towards the interruption of transmission and eventual elimination of schistosomiasis. Making this transition requires a reframing of current control strategies. In this essay, we set out how bridging the gap between recognition of the burden and effective action requires not only medical interventions, but also changes in policy, economics and human behaviour.

If these children stood hand in hand, they would form a human chain long enough to circle the Earth more than three times.

Schistosomiasis is caused by parasitic flatworms of the genus *Schistosoma*, which have co-evolved with humans over millennia. Human infection occurs through contact with freshwater contaminated with parasite larvae that are released from infected snails (Fig. 1). After penetrating the skin, the larvae mature into long-lived adult worms (1–3 cm long) that can survive for decades within blood vessels and produce large numbers of eggs. While some eggs leave the body and sustain transmission, many become trapped in tissues. Species differ in their anatomical localisation and route of egg excretion, giving rise to the distinct clinical syndromes of intestinal and urogenital schistosomiasis. We have found up to 1000 eggs in a single 10 ml urine sample from an *S. haematobium*-infected child. Detection of eggs in stool or urine, therefore, remains a cornerstone of diagnosis.

**Figure 1:**
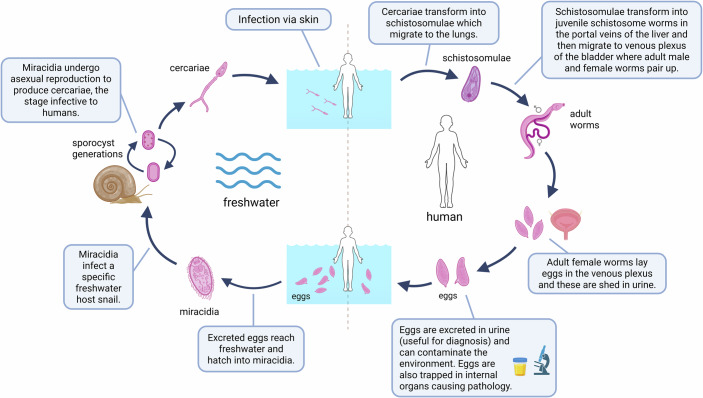
Life cycle of *S. haematobium.* Simplified life cycle of *S. haematobium*, illustrating transmission between humans and freshwater snails, parasite maturation in the human host, and egg release into the environment. Intestinal schistosomes (*S. mansoni* and *S. japonicum*) follow a similar overall cycle but differ in anatomical localisation, route of egg excretion and intermediate host snail species.

## The disease and epidemiology

The epidemiology of schistosomiasis is well understood. There is a distinctive age-related profile, with school-aged children between 5 and 14 years suffering the highest infection prevalence and worm burdens, while adults typically harbour lower parasite loads but accumulate chronic disease over time (WHO, 2020). There is also a substantial but under-recognised burden in pre-school children owing to early-life exposure and missed treatment opportunities.

**Figure 2:**
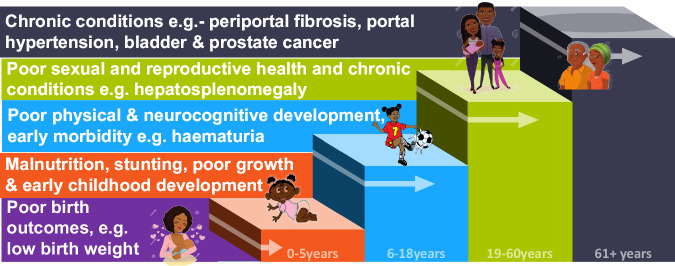
Health consequences of schistosome infection. Lifelong disease course

The health consequences of infection are profound (McManus et al, 2018) (Fig. 2). Following exposure to cercariae, an acute disease (Katayama syndrome) causes fever, with eosinophilia developing about 6 weeks later at the onset of egg laying by mature females, particularly after the first infection. In infants and young children, early exposure compromises early child development and physical health. If untreated, the disease progresses to chronic stages, causing long-term organ damage that closely mimics non-communicable diseases, often obscuring its parasitic origin.

If untreated, the disease progresses to chronic stages, causing long-term organ damage that closely mimics non-communicable diseases, often obscuring its parasitic origin.

**Figure 3:**
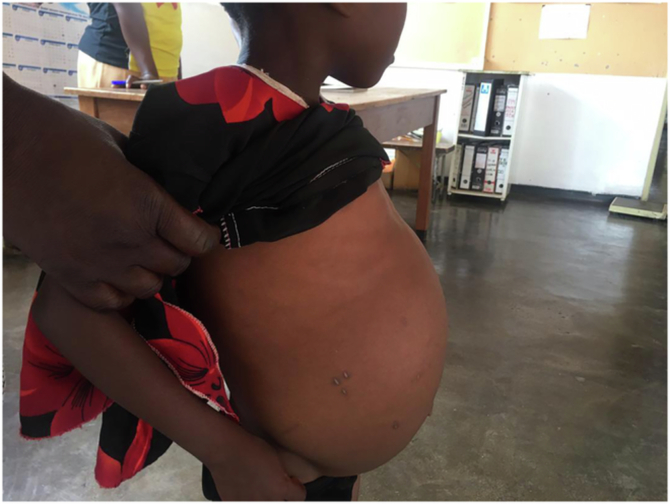
Health consequences of schistosome infection. Pathology (hepatosplenomegaly) in a 5-year-old. Photo credit: Takafira Mduluza, TIBA, University of Zimbabwe.

The intestinal form of the disease, predominantly due to *S. mansoni*, manifests as abdominal pain, diarrhoea and blood in stool, progressing during chronic infection to periportal fibrosis, portal hypertension and hepatosplenomegaly (Fig. 3). Urogenital disease caused by *S*. *haematobium* manifests as haematuria, dysuria and bladder pathology, with long-term risks of hydronephrosis and squamous cell carcinoma of the bladder. In adolescent girls and women, female genital schistosomiasis leads to vaginal and cervical lesions, discharge, dyspareunia, subfertility and increased susceptibility to HIV. In boys and men, genital involvement can affect the prostate and seminal vesicles, contributing to haematospermia and infertility. Across the life course, repeated infection sustains morbidity, with damaging effects on early childhood development, education, and sexual and reproductive health (McManus et al, 2018).

## Schistosome-human co-evolution and immunology

Humans and helminths have co-evolved over millennia, resulting in a delicate immunological relationship. Schistosomes possess sophisticated mechanisms to evade and modulate the host’s immune system, allowing them to survive in the body for years or decades. They cover themselves in host molecules to evade immune detection, continuously renew their surface (the tegument), and secrete immunomodulatory molecules that divert and push the immune response toward a more regulated, anti-inflammatory state. In turn, the host immune system develops parasite-specific partial, non-sterilising immunity so that repeated exposure leads to reduced worm burdens. Generally, the human immune system has evolved to balance protection and regulation, while the parasite has evolved to modulate—but not completely suppress—host immunity (Maizels, 2020). The nature and development of the immune response has long been driven by this host–parasite interaction, and more recently also by anthelminthic treatment, as discussed later.

Human immunity to schistosomes develops slowly over years, driven by cumulative exposure to parasite antigens, particularly those released by dead worms (Colley and Secor 2014). The rate of development of protective immunity is determined by cumulative experience of infection, such that protective immunity develops earlier in areas with higher infection levels. It is now widely accepted that the immune system requires a threshold of parasite antigens for induction (Mitchell et al, 2012). As a consequence, in endemic settings, young children are highly susceptible, while older children and adults progressively develop concomitant immunity—the ability to limit new infections while still harbouring adult worms. Concomitant immunity in human schistosomiasis is characterised by IgE and cytophilic IgG (particularly IgG1/IgG3) mediated protective responses against larval stages. Natural immunity also manifests as a reduction in parasite fecundity mediated by IgA and IgG antibody subclasses.

… in endemic settings, young children are highly susceptible, while older children and adults progressively develop concomitant immunity—the ability to limit new infections while still harbouring adult worms.

Immunologically, protection is associated with a shift from early mixed Th1/Th2 responses toward Th2-dominated and effector responses, including parasite-specific IgE, IgA, IgG1, IgG3, eosinophil activation and antibody-dependent cellular cytotoxicity, alongside regulation by IgG4 and regulatory T cells (McManus et al, 2018). The balance between effector and regulatory responses is critical: effective immunity reduces both the fecundity of established infections and the level of reinfection. The anti-fecundity effects mean a reduction in pathology and in transmission rate, by reducing the number of eggs produced and their hatchability. The slowly developing nature of anti-schistosome immunity, shaped by host age and exposure history, has given rise to the characteristic immuno-epidemiological patterns observed in endemic populations throughout the world.

## Treatment

**Figure 4:**
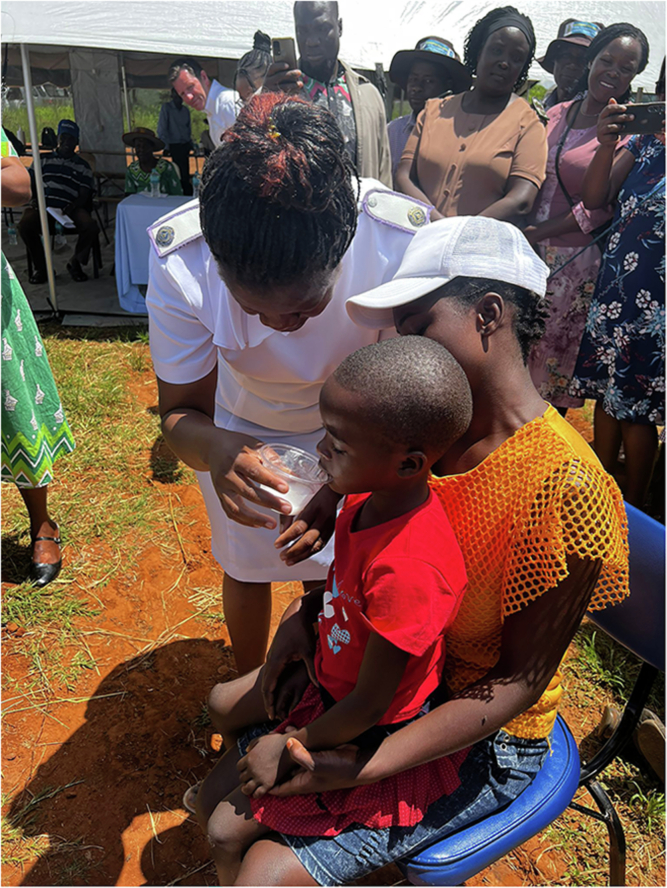
The first child to be treated with arPZQ in Zimbabwe. Pre-school child in Zimbabwe receiving arPZQ for the treatment of *S. haematobium* following diagnosis of infection in January 2026. Photo credit: Helmi Hietanen, TIBA, University of Edinburgh.

Treatment and control of human schistosomiasis are centred on chemotherapy combined with public health interventions. The drug of choice is the anthelminthic drug praziquantel (PZQ), which is effective against the adult stages of all major *Schistosoma* species and is typically given as a single oral dose calibrated to body mass (Fig. 4). It kills only adult worms, and repeated treatments are therefore needed due to reinfection or the presence of immature parasites at the time of administration.

At the population level, schistosomiasis control relies heavily on preventive chemotherapy through mass drug administration (MDA), whereby praziquantel is periodically distributed to at-risk groups without diagnosis—especially school-aged children—in endemic areas. MDA achieves cure rates of 70–90%, with a reduction in egg output typically exceeding 85%. Such programmes have been increasingly implemented with up to 1.3 billion treatments delivered in Africa during the past two decades (WHO, 2025).

The WHO has set the target of achieving elimination of schistosomiasis as a public health problem—that is, reducing the prevalence of heavy infections below 1%—in all 78 endemic countries by 2030, coupled with interruption of transmission in at least 25 countries (WHO, 2020). MDA has already led to substantial reductions in heavy infections, morbidity and prevalence in many endemic countries (WHO, 2025).

Nonetheless, transmission persists in “hotspots”—focal populations where infection prevalence and worm burden remain high despite repeated high-coverage MDA. These hotspots are caused by several transmission-related factors that include continued contact with infective water, poor water and sanitation provision facilitating environmental contamination, and persistent snail populations that maintain transmission (Mduluza et al, 2025).

Furthermore, treatment outcomes are variable, with PZQ having reduced efficacy in some individuals. Initially, this observation sparked concerns over drug resistance, as has been observed in the drug oxamniquine (OXAM) used to treat *S. mansoni* infections. OXAM resistance in *S. mansoni* was first reported in Brazil after its widespread use in control programmes in the 1970s, following years of repeated use in national control programmes. However, we have shown that PZQ efficacy has remained unchanged during four decades of use and that, to date, there is no confirmed, clinically established PZQ resistance to *S. mansoni* or *S. haematobium* in human infections (Fukushige et al, 2021).

Variable treatment outcomes may instead be linked to host pharmacokinetics rather than parasite resistance. PZQ is extensively metabolised in the liver by cytochrome P450 enzymes, and genetic variation in these enzymes can lead to rapid drug metabolism, reducing systemic drug exposure. In such individuals, PZQ concentrations may fail to reach or sustain therapeutic levels required for optimal parasite killing, resulting in reduced cure rates and apparent treatment failure despite drug susceptibility (Zdesenko and Mutapi 2020). There is ongoing research to work out how best to strike a balance between increasing PZQ dosing while minimising the side effects. Beyond pharmacokinetics, experimental studies also show that PZQ efficacy is partly dependent on a competent host immune system, with host antibodies and cellular responses acting synergistically with drug-induced tegumental damage to enhance parasite killing.

MDA over the past two decades has focused primarily on school-aged children, largely excluding children under five years old—who were only formally included in WHO treatment guidelines in 2012. This gap is now being addressed with expanded policy inclusion and the recent availability of a paediatric PZQ formulation, arpraziquantel (arPZQ). Early treatment of schistosomiasis is critical because pathology is driven by immune responses to parasite eggs. In young children, killing adult worms halts egg production and reverses some of the damage, whereas in adults, prolonged egg deposition has already led to irreversible chronic pathology.

In young children, killing adult worms’ halts egg production and reverses some of the damage, whereas in adults, prolonged egg deposition has already led to irreversible chronic pathology.

## Immune-mediated effects of PZQ treatment

By killing adult worms, PZQ does more than clear infection: it also reshapes the host–parasite immune relationship. Schistosomes survive for years partly by inducing regulatory immune responses in the host that predominate over protective effector responses, consequently dampening anti-parasite immunity. Over decades, cumulative antigen exposure gradually promotes stronger effector responses that reduce parasite fecundity and susceptibility to reinfection.

**Figure 5:**
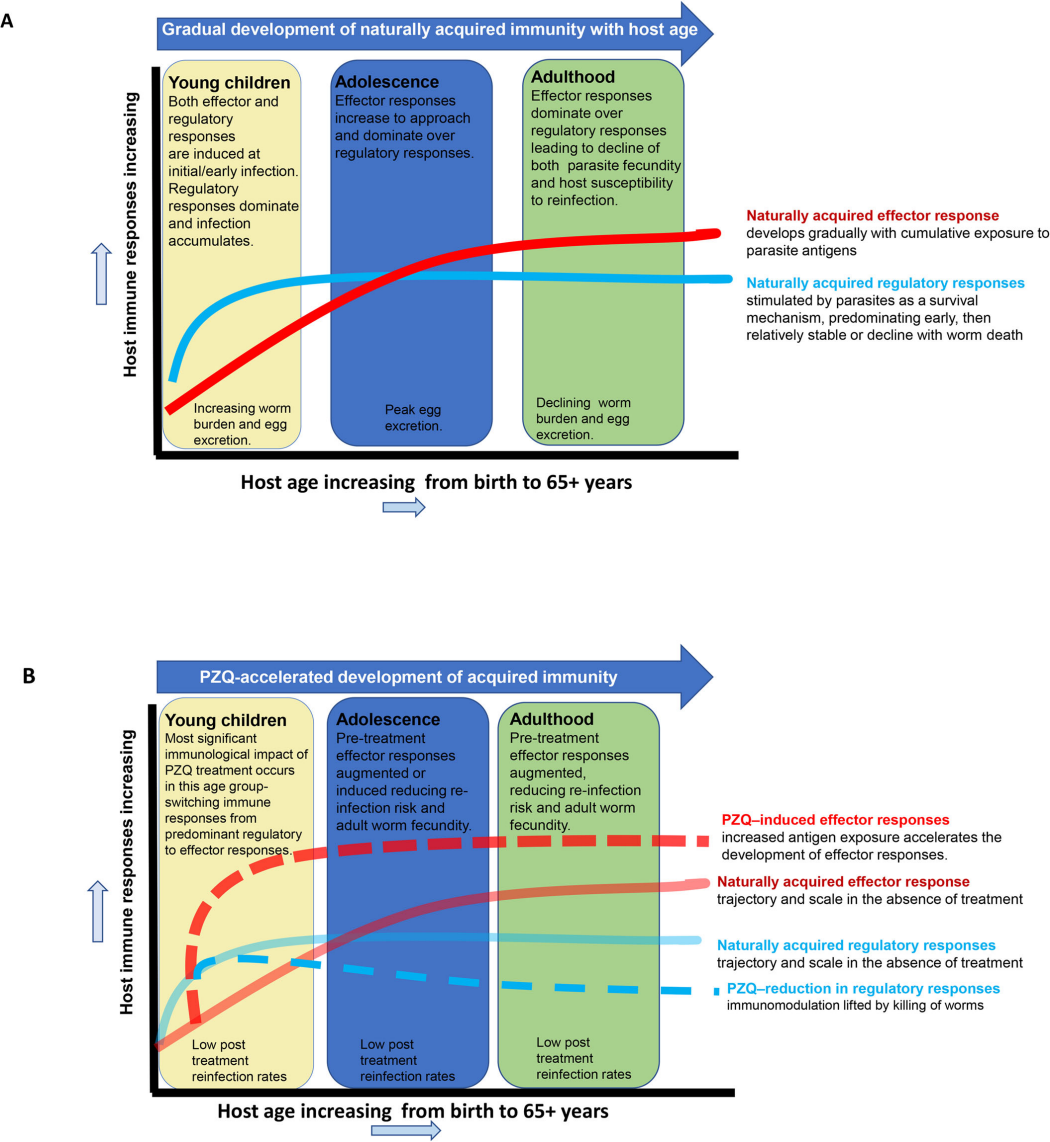
Simplified model of the development of natural vs PZQ-induced protective immunity in humans. Conceptual model of immune maturation in human schistosomiasis showing the switching of immune phenotype from susceptible to partial immunity in affected people. (**A**) Naturally acquired immunity develops gradually with cumulative parasite antigen exposure, and protective effector responses (immune phenotype) emerge slowly over decades. (**B**) PZQ treatment accelerates this process by increasing antigen exposure through worm death and by removing parasite-induced immune suppression, promoting earlier development within weeks of protective anti-parasite responses.

PZQ accelerates this natural trajectory through two complementary mechanisms. First, dead worms release a large amount of parasite antigens (Harnet and Kusel, 1986) that boost schistosome-specific immune responses. In endemic populations, treatment is associated with increased parasite-specific antibody levels, antibody class switching, and changes in cytokine and T-helper cell profiles (Mutapi et al, 1998; Mitchell et al, 2012). Second, killing living adult worms relieves parasite-mediated immunosuppression, which shifts the balance from a predominantly regulatory toward a more protective immune phenotype. This switch occurs within weeks, effectively accelerating a process that naturally takes decades to occur. These treatment-induced changes are associated with lower reinfection rates and are amplified by repeated treatment, forming the basis of the proposed infection–treatment–immunity paradigm for schistosomiasis (Fig. 5; Mutapi et al, 2013). Similar principles underpin successful infection-and-treatment vaccination strategies used in veterinary medicine, such as the *Theileria parva* East Coast fever vaccine. The conceptual relationship between naturally acquired and PZQ-augmented immunity is illustrated in Fig. 5.

The immunological impact of killing worms can also ‘spill over’ to unrelated antigens. Several studies in different human populations show that PZQ treatment reverses schistosome-induced immunosuppression to unrelated antigens, such as vaccines, leading to increased cellular and antibody reactivity (Nono et al, 2022). Studies in Africa have confirmed that responses to BCG, tetanus and measles vaccines in helminth-infected individuals are augmented by deworming. Nonetheless, despite this enhancement at the immunological level, the improved responses do not seem to translate to clinically observable health benefits. To date, there are no epidemiological data showing improved vaccine effectiveness in terms of reduction of incidence, prevalence or disease severity—attributable to schistosome treatment of African populations.

Other bystander antigens affected by anthelminthic treatment are allergens and autoantigens (Maizels, 2020). The hygiene hypothesis posits that reduced exposure to infectious agents, particularly helminths, in early life leads to increased susceptibility to allergic and autoimmune diseases due to insufficient immune regulation. In the case of schistosomiasis, immunological studies show that the killing of worms by PZQ removes parasite‑induced immunosuppression, leading to increased immune reactivity/sensitisation to both allergens and autoantigens. With the advent of large-scale distribution of PZQ, concerns were raised that removing the parasite-immune regulation would lead to increases in allergic and autoimmune diseases in populations receiving MDA. However, to date, there have been no reports of detectable increases in allergic or autoimmune disease in areas undergoing MDA for schistosomiasis in the past 20 years.

Several explanations have been proposed for this observation. The first is that PZQ does not eliminate exposure entirely; in endemic settings, individuals are frequently re-exposed, maintaining a degree of immune regulation. Although this is the case in some people, it is not sufficient to explain population-wide effects. More likely is the second reason, which is related to evolutionary trade-offs in immune-resource allocation. Immune responses after treatment tend to recalibrate rather than overshoot, stabilising at a new equilibrium rather than shifting toward pathological hyperreactivity. Thus, treatment does not abruptly remove immune regulation but instead shifts the host–parasite relationship along a continuum. Together, these findings highlight that PZQ treatment acts not only as an antiparasitic drug but also, by releasing antigens from killed worms, as an immunological modulator, accelerating protective immunity without triggering the feared collateral rise in immune-mediated disease.

… PZQ treatment acts not only as an antiparasitic drug but also, by releasing antigens from killed worms, as an immunological modulator, accelerating protective immunity…

## Paediatric schistosomiasis treatment

The finding that PZQ treatment accelerates the development of protective immunity has important implications for treatment policy. For the past two decades, the focus on school-aged children meant that pre-school-aged children (PSACs, children 5 years old and below) were overlooked because they were wrongly assumed to be at low risk of infection and disease, and because child-appropriate drug formulations, dosing guidance and safety data were initially unavailable. Another concern was that their immune systems might be too immature to mount the parasite-specific responses that work together with PZQ in killing the adult worms.

Our studies and those of others, have shown that these assumptions were incorrect. Young children are immunologically capable of generating the antibody and cellular responses that synergise with PZQ, achieving high cure rates. Importantly, early treatment of PSACs also accelerates the development of protective immunity before substantial pathology develops. Epidemiological studies have demonstrated that PSACs already harbour adult worms and can suffer clinically significant morbidity. Treating them therefore removes the immediate source of disease while simultaneously accelerating the development of immunity protective against reinfection, providing an additional rationale for early intervention.

The development of the paediatric praziquantel formulation arPZQ, removed the final technical barrier to fully integrating PSAC into schistosomiasis control programmes. The remaining challenges are operational: ensuring equitable access and delivery through sustainable financing and strengthened health systems.

## Beyond PZQ treatment: interrupting transmission

While large-scale treatment programmes have reduced the prevalence of schistosomiasis with substantial declines in levels of infection and morbidity during the past 25 years, these gains have not been accompanied by the sustained interruption of transmission. Reinfection remains common, particularly in communities where daily contact with infested water is unavoidable. The immediate barrier to interrupting transmission is not a lack of knowledge, but a gap between what is needed and what is done at scale. It is clear that effective control requires integrated approaches beyond drug treatment. Comprehensive strategies must include improved water, sanitation and hygiene (WASH), health education, behaviour change and snail control. In some settings, veterinary interventions targeting animal reservoirs are also needed.

The immediate barrier to interrupting transmission is not a lack of knowledge, but a gap between what is needed and what is done at scale.

To implement these, the economic and behavioural factors facilitating continued transmission must be addressed. First, the health impact of schistosomiasis must be recognised and resources allocated to infection prevention. Studies from Tanzania and Uganda show that schistosomiasis is often normalised, with symptoms such as haematuria being taken as a sign of approaching adulthood. This leads communities to concentrate on acute diseases such as malaria in their health-seeking behaviour and out-of-pocket spending on interventions. Our studies and others indicate that affected people do not prioritise investment in WASH infrastructure when allocating family resources; for example, preferring to invest in mobile phones rather than constructing toilets or wells.

Improved WASH reduces domestic and sanitation-related exposure, but it does not entirely eliminate contact with infested water. In many endemic settings, such contact is integral to livelihoods and daily life through farming, fishing, domestic use and recreation and is unlikely to change rapidly. Behavioural factors further sustain transmission: open defaecation persists even where sanitation exists, reflecting social norms and preferences. As a result, while WASH is essential, it is unlikely to be sufficient on its own. This underscores the need to make unavoidable water contact safer; either by targeting snails and parasite stages in the water, or by reducing human susceptibility to infection and transmission potential.

Environmental control options exist but are difficult to sustain at scale. Molluscicides such as niclosamide can reduce snail populations and cercariae, but their application in large water bodies is costly, logistically complex, environmentally sensitive and often short-lived due to rapid reinfestation. Biological control approaches using molluscivorous fish, ducks or prawns are similarly constrained by local effectiveness, ecological risks and the need for ongoing management. Locally, such interventions can be effective, but only as part of a comprehensive program to reduce transmission.

## Political will and structural realities

Several countries—including Japan, Morocco and Tunisia—have demonstrated that elimination is possible. Their success required sustained, integrated approaches combining treatment, environmental modification, snail control, WASH, surveillance and strong political commitment.

In contrast, sub-Saharan Africa has relied predominantly on periodic MDA. This reflects structural and financial realities rather than evidence gaps: MDA is inexpensive, scalable and has to date been financed by donors, whereas interrupting transmission requires long-term, multisectoral investment in infrastructure and environmental change. Even if MDA reduces morbidity, persistent exposure sustains reinfection, and chronic disease remains unaddressed.

In much of sub-Saharan Africa, the de-prioritisation of schistosomiasis by individuals is reflected at the political level, such that control has not seen major national investments beyond largely donor-funded MDAs. There are limited disease management programmes at primary and tertiary health centres and consequently a dearth of diagnostics, treatments and care pathways in most endemic regions.

Elevating the prioritisation of schistosomiasis requires more than health education, even though that is essential. It needs to be embedded in the development agenda. In addition to the substantial health benefits, the economic benefits from controlling the infection need to be quantified and highlighted. Successful interventions can and should be used to demonstrate the return on investment for affected individuals, policymakers and ministries of health and finance.

## Context-specific solutions

Accelerating progress on schistosomiasis requires approaches aligned with local realities. Because changing large-scale infrastructure and behaviour takes time, there is a need to complement them with strategies that quickly reduce the health consequences of exposure. Host-directed interventions offer a promising pathway. Prophylactic drug regimens, modelled on malaria prevention strategies, could reduce the establishment of new infections during high-risk periods, using combinations of praziquantel and drugs targeting immature parasite stages. Emerging approaches aim to block infection at the skin through topical formulations that prevent cercarial penetration, offering immediate protection without requiring behavioural change (Ingram et al, 2002).

Vaccination represents a longer-term solution. An anti-infection vaccine would make water contacts safe, while an anti-transmission vaccine would reduce contamination of rivers with schistosome eggs. To date, there is no human vaccine against schistosomes, although the need has long been recognised since the 1950s. While several candidates—including the Sm14, Sm-TSP-2, Sh28GST and Sm-p80 antigens—have reached clinical evaluation, progress has been limited by the parasite’s complex, multiple life stages, its immunomodulatory properties, and reliance on single-antigen designs (Molehin et al, 2022). A broadly protective, multivalent vaccine could complement—and ultimately reduce reliance on—MDA. Together, these strategies shift the emphasis from preventing water contact to making contact safer, aligning control efforts with how people actually live and opening more realistic pathways to elimination.

**Figure 6:**
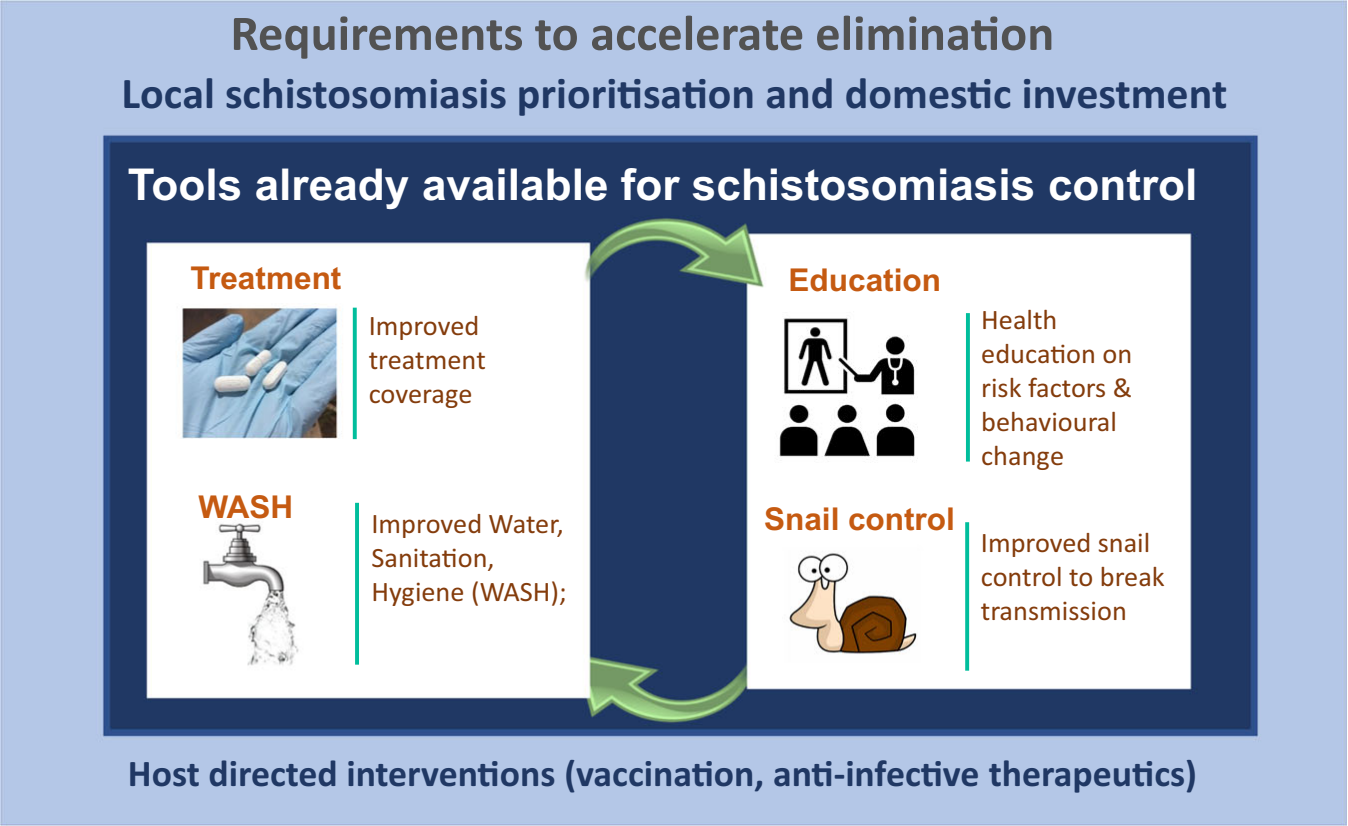
Integrated approach to schistosomiasis elimination. Schistosomiasis control currently relies on the available tools: preventive chemotherapy with PZQ, improving WASH and snail control, health education and surveillance. But elimination requires a shift to integrated, system-strengthening approaches supported by domestic financing and by better host-directed tools such as vaccines and anti-infective therapies aligned with the lived realities of affected communities.

Schistosomiasis will not be eliminated by relying on a single tool applied uniformly across diverse settings. This disease endures because it is embedded in the conditions of daily life, thriving where poor WASH, poverty and weak health systems intersect. Decades of experience indicate that, as long as those conditions remain unchanged, transmission will continue. Meeting internationally agreed targets for elimination and interruption of transmission requires a fundamental shift—from short-term control to long-term transformation. This means integrating scientific knowledge with development, technology with behaviour, and innovation with the lived realities of affected communities; all supported by domestic financing and health systems strengthening (Fig. 6).

Schistosomiasis will not be eliminated by relying on a single tool applied uniformly across diverse settings.

The WHO Neglected Tropical Diseases (NTD) Roadmap’s Pillar 3 is focused on changing operating models and culture to facilitate country ownership of NTD control programmes. This encourages endemic countries to move beyond fragmented, donor-driven models and invest in coordinated, multisectoral strategies to address the root causes of disease transmission. Crucially, endemic communities must be at the centre of these efforts—fully informed, actively engaged and empowered to shape and adopt interventions. The WHO’s goal of eliminating schistosomiasis as a public health problem by 2030 may prove too ambitious, but there has been progress, and elimination is feasible in the longer term, given the will and a concerted effort to deliver it.

## Supplementary information

Peer Review File

## References

[CR1] Colley DG, Secor WE (2014) Immunology of human schistosomiasis. Parasite Immunol 36:347–357 25142505 10.1111/pim.12087 PMC4278558

[CR2] Fukushige M, Chase-Topping M, Woolhouse MEJ, Mutapi F (2021) Efficacy of praziquantel has been maintained over four decades (from 1977 to 2018): a systematic review and meta-analysis of factors influence its efficacy. PLoS Negl Trop Dis 15:e0009189 33730095 10.1371/journal.pntd.0009189 PMC7968639

[CR3] Harnett W, Kusel JR (1986) Increased exposure of parasite antigens at the surface of adult male *Schistosoma mansoni* exposed to praziquantel in vitro. Parasitology 93:401–405 2431374 10.1017/s0031182000051568

[CR4] Ingram RJ, Bartlett A, Brown MB, Marriott C, Whitfield PJ (2002) Dimethicone barrier cream prevents infection of human skin by schistosome cercariae: evidence from Franz Cell studies. J Parasitology 88:399–402 10.1645/0022-3395(2002)088[0399:DBCPIO]2.0.CO;2 12054019

[CR5] Maizels RM (2020) Regulation of immunity and allergy by helminth parasites. Allergy 75:524–534 31187881 10.1111/all.13944

[CR6] McManus DP, Dunne DW, Sacko M et al (2018) Schistosomiasis. Nat Rev Dis Primers 4:13 30093684 10.1038/s41572-018-0013-8

[CR7] Mduluza T, Zdesenko G, Tagwireyi P, Jones CM, Mutapi F (2025) Identifying hotspots of *S. haematobium* infection following praziquantel treatment during multiple annual mass drug administration campaigns in Zimbabwe. PLoS Negl Trop Dis 19:e0013546 40991670 10.1371/journal.pntd.0013546 PMC12520393

[CR8] Mitchell KM, Mutapi F, Savill NJ, Woolhouse ME (2012) Protective immunity to Schistosoma haematobium infection is primarily an anti-fecundity response stimulated by the death of adult worms. Proc Natl Acad Sci USA 109:13347–13352 22847410 10.1073/pnas.1121051109 PMC3421178

[CR9] Molehin AJ, McManus DP, You H (2022) Vaccines for human schistosomiasis: recent progress, new developments and future prospects. Int J Mol Sci 23:2255 35216369 10.3390/ijms23042255 PMC8879820

[CR10] Mutapi F, Billingsley PF, Secor WE (2013) Infection and treatment immunizations for successful parasite vaccines. Trends Parasitol 29:135–141 23415733 10.1016/j.pt.2013.01.003 PMC3884123

[CR11] Mutapi F, Ndhlovu PD, Hagan P, Spicer JT, Mduluza T, Turner CM, Chandiwana SK, Woolhouse ME (1998) Chemotherapy accelerates the development of acquired immune responses to *Schistosoma haematobium* infection. J Infect Dis 178:289–293 9652458 10.1086/517456

[CR12] Nono JK, Kamdem SD, Musaigwa F, Nnaji CA, Brombacher F (2022) Influence of schistosomiasis on host vaccine responses. Trends Parasitol 38:67–79 34389214 10.1016/j.pt.2021.07.009

[CR13] World Health Organization (2020) Ending the neglect to attain the sustainable development goals: a road map for neglected tropical diseases 2021–2030. World Health Organization, Geneva

[CR14] World Health Organization (2025) Global report on neglected tropical diseases 2025. World Health Organization, Geneva

[CR15] Zdesenko G, Mutapi F (2020) Drug metabolism and pharmacokinetics of praziquantel: a review of variable drug exposure during schistosomiasis treatment in human hosts and experimental models. PLoS Negl Trop Dis 14:e0008649 32976496 10.1371/journal.pntd.0008649 PMC7518612

