# Supplementary material for: The big picture: Parasites, People and the Path to Ending Schistosomiasis
Source: EMBO Rep. 2026 Jun 26;27(14):3895–902. doi: 10.1038/s44319-026-00845-x (PMC13400727; doi:10.1038/s44319-026-00845-x)
Supplement: Supplementary file 1 — Peer Review File [file 44319_2026_845_MOESM1_ESM.pdf]

EMBO reports

The Big Picture: Parasites, People, and the Path to Ending Schistosomiasis

Francisca Mutapi and Mark Woolhouse

Corresponding author: Francisca Mutapi (f.mutapi@ed.ac.uk)

Review Timeline:

Submission Date:	12th May 26
Accepted:	11th Jun 26

Editor: Holger Breithaupt

Transaction Report:

This article was editorially reviewed.
